# Influence of Colloidal Au on the Growth of ZnO Nanostructures

**DOI:** 10.3390/nano11040870

**Published:** 2021-03-29

**Authors:** Frank Güell, Andreu Cabot, Sergi Claramunt, Ahmad Ostovari Moghaddam, Paulina R. Martínez-Alanis

**Affiliations:** 1 ENFOCAT-IN2UB, Universitat de Barcelona (UB), C/Martí i Franquès 1, 08028 Barcelona, Catalunya, Spain; frank.guell@ub.edu (F.G.); sclaramunt@el.ub.edu (S.C.); 2 Catalonia Institute for Energy Research (IREC), Sant Adrià de Besòs, 08930 Barcelona, Catalunya, Spain; acabot@irec.cat; 3 ICREA, Pg. Lluis Companys 23, 08010 Barcelona, Catalunya, Spain; 4 Department of Materials Science, Physical and Chemical Properties of Materials, South Ural State University, Lenin Ave. 76, 454080 Chelyabinsk, Russia; ostovary@aut.ac.ir

**Keywords:** ZnO nanostructures, Au nanoparticles, VLS, solvents, thermal dewetting

## Abstract

Vapor-liquid-solid processes allow growing high-quality nanowires from a catalyst. An alternative to the conventional use of catalyst thin films, colloidal nanoparticles offer advantages not only in terms of cost, but also in terms of controlling the location, size, density, and morphology of the grown nanowires. In this work, we report on the influence of different parameters of a colloidal Au nanoparticle suspension on the catalyst-assisted growth of ZnO nanostructures by a vapor-transport method. Modifying colloid parameters such as solvent and concentration, and growth parameters such as temperature, pressure, and Ar gas flow, ZnO nanowires, nanosheets, nanotubes and branched-nanowires can be grown over silica on silicon and alumina substrates. High-resolution transmission electron microscopy reveals the high-crystal quality of the ZnO nanostructures obtained. The photoluminescence results show a predominant emission in the ultraviolet range corresponding to the exciton peak, and a very broad emission band in the visible range related to different defect recombination processes. The growth parameters and mechanisms that control the shape of the ZnO nanostructures are here analyzed and discussed. The ZnO-branched nanowires were grown spontaneously through catalyst migration. Furthermore, the substrate is shown to play a significant role in determining the diameters of the ZnO nanowires by affecting the surface mobility of the metal nanoparticles.

## 1. Introduction

ZnO is a direct band-gap (3.37 eV) semiconductor having a large exciton binding energy (60 meV). It is widely used in numerous applications due to the abundance and low cost of its constituent elements and its unique semiconducting, optoelectronic and piezoelectric properties [1]. These functional properties of ZnO can be enhanced at the nanoscale through the growth of nanostructures such as nanotubes (NTs), nanosheets (NSs), and nanowires (NWs) [2]. ZnO NWs show efficient third harmonic UV generation [3] and are valuable materials not only for future nanoscale sensors [4], but also for optoelectronic devices such as solar cells [5]. For all these applications, the availability of ZnO NWs with high-crystal quality is mandatory [6].

ZnO NWs can be synthesized using different approaches such as solution growth [7], wet chemical methods [8], template-induced growth [9] and vapor transport [10]. The vapor-transport method allows growing high-quality single-crystal ZnO NWs by the vapor-liquid-solid (VLS) process using Au as a catalyst. In this process, an Au thin layer deposited over a substrate is annealed to generate Au clusters that act as a catalyst for the ZnO NW growth. The control over the ZnO NW morphology comes from the layer thickness that determines the Au cluster size after the thermal annealing [11].

An alternative strategy to control the morphology of ZnO NWs grown by the VLS process is the use of colloidal Au nanoparticles (NPs) as a catalyst [12]. A direct correlation exists between the diameters of the Au NPs and the resulting ZnO NWs [13], and between the density of deposited Au NPs and the resulting density of ZnO NWs [14,15]. Such parameters have an important impact on the functional properties of the produced material. As an example, lower areal densities improve the field emission characteristics of ZnO NWs [16]. Additional advantages of using colloidal Au NPs as a catalyst are a simplified deposition process and a drastic reduction of the total amount of Au used. Thus, colloidal Au NPs offer the double opportunity to precisely control the properties of the produced ZnO NWs while at the same time significantly cutting down the processing cost. Furthermore, the use of colloidal Au NPs allows a simple and precise selection of the ZnO NW growth location [17,18].

Au NPs supported over a substrate can diffuse, approach and coalescence at high temperature. These processes have been extensively studied [19,20]. However, until now, a thorough study of the influence of the colloid parameters on the morphology of the ZnO nanostructures obtained remains to be explored.

In this work, we study the influence of different colloidal suspension of Au NPs on the growth of single-crystal ZnO nanostructures obtained by a catalyst-assisted growth through the vapor-transport method. Diverse ZnO nanostructures have been obtained as a function of the growth parameters, the solvent used and the concentration of colloidal Au NPs deposited over silica on silicon (SiO_2_/Si) and alumina (Al_2_O_3_) substrates. The ZnO nanostructures have been characterized by field-emission scanning electron microscopy (FESEM), high-resolution transmission electron microscopy (HRTEM) and photoluminescence (PL) spectroscopy.

## 2. Experimental Section

Chemicals were used without additional purification except deionized water. HAuCl_4_ tetrachloroauric acid (HAuCl_4_, 49.0% Au basis, Fluka, Buchs, Switzerland), decanoic acid (DA, 98.0%, Fluka, Buchs, Switzerland), didodecyldimethylammonium bromide (DDAB, 97.0%, Fluka, Buchs, Switzerland), hexadecyltrimethylammonium bromide (CTAB, 98% Fluka, Buchs, Switzerland), sodium borohydride (NaBH_4_, 99%, Sigma-Aldrich, St. Louis, MO, USA), tetrabutylammonium borohydride (TBAB, 99.0%, Fluka, Buchs, Switzerland), zinc oxide powder (ZnO, 99.0%, Sigma-Aldrich, St. Louis, MO, USA), graphite powder (C, 99.99%, Sigma-Aldrich, St. Louis, MO, USA), argon gas (Ar, 99.999%, Sigma-Aldrich, St. Louis, MO, USA), toluene (99.8%, Sigma-Aldrich, St. Louis, MO, USA) and hexane (95%, Sigma-Aldrich, St. Louis, MO, USA).

### 2.1. Au NP Synthesis and Deposition

Colloidal Au NPs in non-polar organic solvents were prepared following a modification of a previously reported procedure [21]. Briefly, an Au salt solution was prepared by dissolving 8.4 mg of HAuCl_4_ in 2.5 mL of a 0.1 M solution of DDAB in toluene by sonication. The resulting solution had an orange-red color. In a separate flask, 43 mg of DA was dissolved in 2.5 mL of toluene. Then, 25 mg TBAB was dissolved in 1 mL of a 0.1 M solution of DDAB in toluene, which was mixed with the DA solution. Subsequently, the Au solution was injected into the TBAB and DDAB solution under vigorous stirring. Just after the injection of the Au salt, the solution color changed to deep red, indicating the formation of Au NPs. Colloidal Au NPs in water were prepared following a previously reported procedure using CTAB as a surfactant, water as a solvent and NaBH_4_ as a reducing agent [22].

Au NPs were purified using multiple centrifugation and re-dispersion steps and were finally suspended at concentrations of 6·10^12^, 6·10^11^, and 1.2·10^11^ NPs·cm^−3^ in different solvents such as water, toluene, and hexane. Then, they were deposited over the substrates by drop-casting at room temperature.

The morphology of the colloidal Au NPs was characterized using TEM with a Philips CM-30 (300 kV), Eindhoven, Holland. For TEM characterization, samples were prepared by placing a drop of the colloidal solution containing the Au NPs onto a carbon-coated copper grid at room temperature and ambient atmosphere.

### 2.2. ZnO Nanostructures Growth and Characterization

The Au-catalyzed VLS process was used to grow ZnO nanostructures over the amorphous surface of SiO_2_/Si and Al_2_O_3_ substrates. Powders of ZnO and C in a proportion 1:1 were mixed and the synthesis was carried out in a horizontal quartz tube placed in a chemical vapor deposition (CVD) furnace. The standard growth parameters were a temperature of 900 °C, pressure at 760 Torr, and Ar gas flow at 400 sccm.

The structural characterization of the ZnO nanostructures was performed by FESEM and HRTEM using a Hitachi H-4100FE, Tokio, Japan and a Jeol 12010F, Tokio, Japan, respectively. The room temperature PL measurements were performed using a chopped Kimmon IK Series He-Cd laser, Tokio, Japan (325 nm and 40 mW). Fluorescence was dispersed with an Oriel, Irvine, California, USA Cornerstone 1/8 74,000 monochromator, detected with a Hamamatsu, Japan R928 photomultiplier, and amplified through a Stanford Research Systems, Sunnyvale, California, USA SR830 DSP. A filter in 360 nm was used to stray light, and all the spectra were corrected for the response function of the setups.

## 3. Results and Discussion

### 3.1. Effect of Growth Parameters

**Figure 1:**
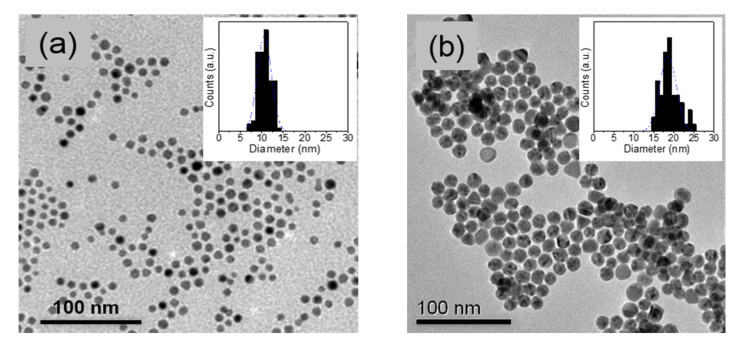
TEM micrographs of the colloidal Au NPs in (**a**) toluene and (**b**) water solvents and corresponding size histogram.

Controlling the growth parameters for the VLS process is essential to be able to tune the morphology and crystal quality of the produced ZnO NWs [23]. Figure 1 shows representative TEM micrographs of the colloidal Au NPs obtained in non-polar organic solvents and water, and used as a catalyst. The colloidal Au NPs grown in non-polar organic solvents had an average diameter of 11 ± 1 nm (see the histogram inset to Figure 1). The Au NPs grown in water were slightly larger, with an average diameter of 19 ± 3 nm.

A drop of a 6·10^12^ NPs·cm^−3^ suspension of Au NPs in water was placed over a SiO_2_/Si substrate. The ZnO/C powder mixture was located in the CVD furnace together with the seeded SiO_2_/Si substrate and the synthesis was carried out. Three parameters were modified in this study to see their effect on the morphology of the ZnO NWs synthesized: growth temperature, growth pressure, and Ar gas flow.

**Figure 2:**
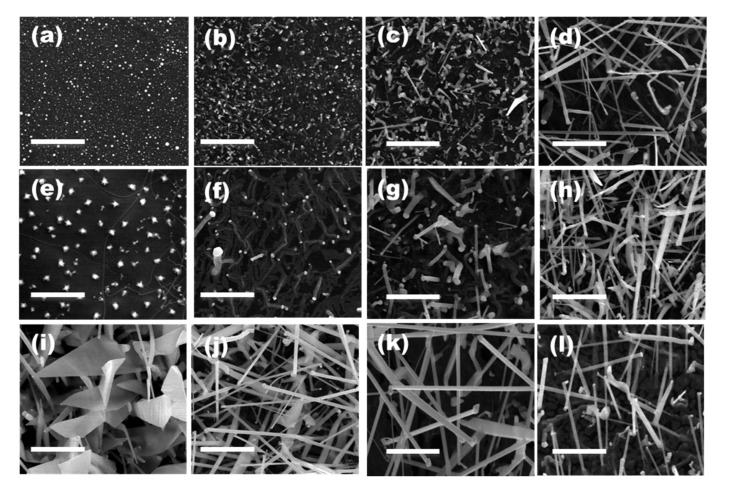
FESEM images of the samples synthesized over SiO_2_/Si substrates using water solvent at (**a**–**d**) different growth temperatures of 700, 800, 850, and 900 °C; (**e**–**h**) different growth pressures of 76, 200, 380, and 600 Torr; and (**i**–**l**) different Ar gas flows of 50, 100, 200, and 600 sccm, respectively All scale bars are 2 µm.

First, the growth temperature effect in the morphology of the ZnO NWs synthesized over the SiO_2_/Si substrates was studied. Samples 1 to 4 were grown at temperatures of 700, 800, 850, and 900 °C, while keeping pressure at 760 Torr and the Ar gas flow at 400 sccm (Table 1). The obtained samples were analyzed by FESEM to trace the morphological evolution (Figure 2a–d). For samples processed at 800 °C and lower temperatures, no ZnO NWs were grown and only Au nanoclusters were found on the SiO_2_ surface (see Figure 2a,b). This result is explained taking into account that the decomposition of the ZnO with C takes place at around 850 °C [24]. The growth of ZnO NWs was achieved when increasing the temperature to 850 °C, as shown in Figure 2c. Note that ZnO NWs did not grow vertically with respect to the substrate but randomly oriented due to the lack of lattice compatibility between the ZnO and the amorphous SiO_2_ surface. This random orientation was also previously observed in ZnO NWs grown using sputtered Au thin layers as catalyst over SiO_2_/Si substrates [23].

The average diameter of the produced ZnO NWs (ca. 100 nm) was significantly larger than that of the colloidal Au NPs used as a catalyst (19 nm). During the synthesis process, the high temperatures forced the diffusion and coalescence of the deposited Au NPs [20], which resulted in the formation of bigger Au domains, see Figure 2a. During the ZnO synthesis, these Au domains are alloyed with the Zn vapor coming from the carbothermal reduction of the ZnO/C powder mixture, and when the Au is supersaturated of Zn, the Zn is oxidized at the liquid–solid interface of the domains. Then, the nucleation and growth of the solid ZnO NWs with larger diameters takes place. Moreover, ZnO NWs grow uniformly distributed over the SiO_2_/Si substrate due to the uniform distribution of the colloidal Au NPs over the SiO_2_ surface (see Figure 2d).

Second, the growth pressure effect in the morphology of the ZnO NWs synthesized over the SiO_2_/Si substrates was studied. Samples 5 to 8 were grown at pressures of 76, 200, 380, and 600 Torr, while keeping the temperature at 900 °C and the Ar gas flow at 400 sccm—see Table 1. FESEM images of the samples 5 and 6 grown at 76 and 200 Torr, respectively, show only Au particles on the SiO_2_ surface (see Figure 2e,f). By increasing the pressure (380 Torr), the growth of ZnO NWs was achieved, as shown in Figure 2g. Figure 2h shows the growth of ZnO NWs at 600 Torr. As the carbothermal reduction reaction of the ZnO/C powder mixture is temperature dependent, the pressure strongly affects the supersaturation level of the Zn vapor in the Au [25]. The Zn vapor concentration is higher at lower pressures in the tube furnace, and the Zn atoms are not alloyed with Au resulting in no ZnO NW growth [26].

Third, the effect of the Ar gas flow on the morphology of ZnO NWs grown over the SiO_2_/Si substrates was studied. Samples 9 to 12 were synthesized at different Ar gas flows of 50, 100, 200, and 600 sccm, while keeping temperature at 900 °C and pressure at 760 Torr (see Table 1). At Ar gas flows lower than 100 sccm, the growth process generated two-dimensional nanostructures with triangular shape, ZnO NSs (see Figure 2i), while at higher Ar gas flows than 100 sccm, ZnO NWs were obtained, as shown in Figure 2j–l. The Ar gas flow seemed to have the most dramatic effect in the ZnO nanostructure morphology. This observation is explained by the strong effect that the Ar gas flow has on the concentration of Zn, and thus on the deposition rate, which is critical to determine the type of growth that occurs. The deposition rate is higher at low Ar gas flows, which transport higher concentrations of Zn and thus allow for secondary ZnO nucleation. In this scenario, under a certain supersaturation, the competition between different surface planes to capture impinged molecules determines the final morphology of the ZnO NWs or NSs [27]. Moderately high supersaturation may create a low-surface-energy tip, which allows molecules to diffuse away easily to energetic side surfaces causing the growth of ZnO NSs.

### 3.2. Solvent and Concentration Effect

In the following, the standard growth parameters—temperature at 900 °C, pressure at 760 Torr, and Ar flow at 400 sccm—were used to study the influence of the Au colloid solvent and concentration on the catalyst-assisted growth of the ZnO NWs. Colloidal Au NPs in water, toluene and hexane were deposited at concentrations of 6·10^12^, 6·10^11^ and 1.2·10^11^ NPs·cm^−3^ over SiO_2_/Si substrates, and the synthesis were carried out at the standard growth parameters—see Table 2. The main difference between solvents is the slightly different size and surface chemistry of the particles in water compared with those in toluene and hexane, and the different evaporation rate of the three solvents, water being the slowest to be evaporated and hexane the fastest, thus resulting in different Au NP distributions on the substrate.

**Figure 3:**
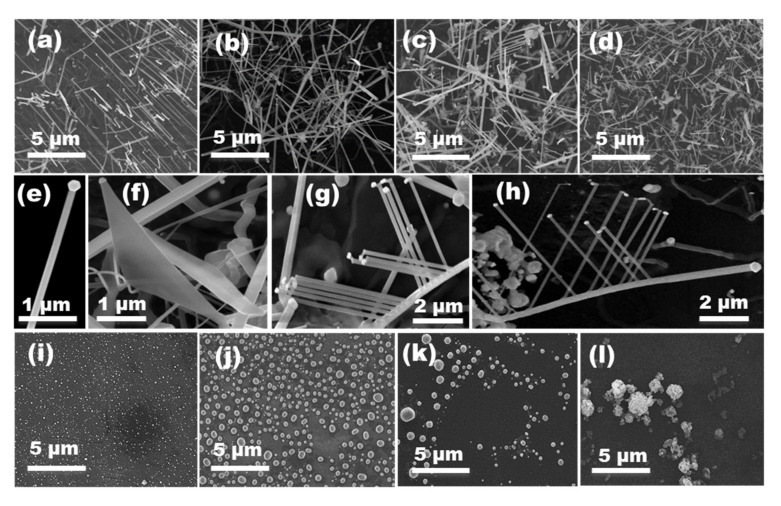
FESEM images of the ZnO NWs grown over SiO_2_/Si substrates using (**a**) water and (**b**) toluene solvents for the colloidal deposition at 6·10^16^ NPs·cm^−3^ concentration, and (**c**,**d**) using toluene solvent for the colloidal depositions at 6·10^15^ and 1.2·10^15^ NPs·cm^−3^ concentrations, respectively. (**e**–**h**) ZnO nanostructures obtained with different morphologies over the SiO_2_/Si substrates. FESEM images of the dewetted colloidal Au NPs deposited over the SiO_2_/Si substrates after the annealing at 900 °C using (**i**) water and (**j**) toluene solvents for the colloidal deposition at 6·10^16^ NPs·cm^−3^ concentration, and (**k**,**l**) using toluene solvent for the colloidal depositions at 6·10^15^ and 1.2·10^15^ NPs·cm^−3^ concentrations, respectively.

**Figure 4:**
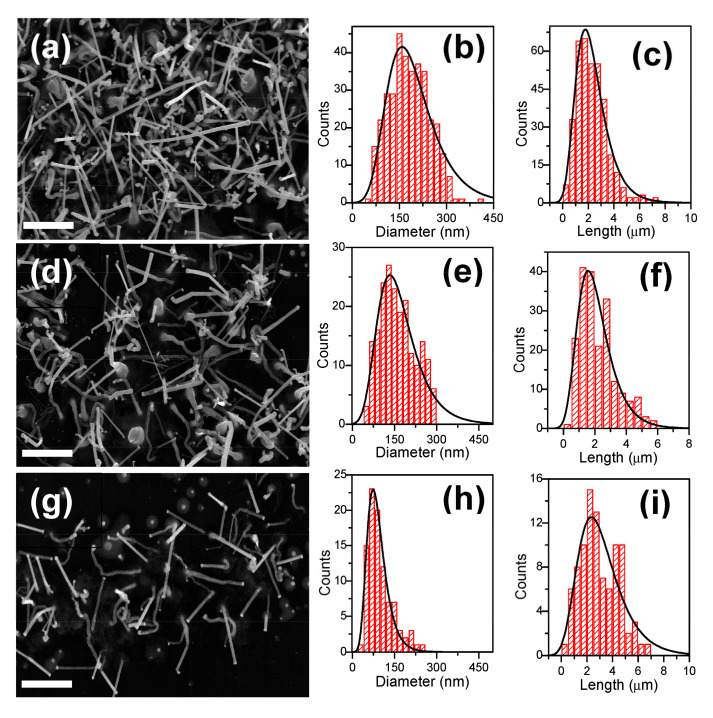
FESEM images of the ZnO NWs grown over SiO_2_/Si substrates using hexane solvent for the colloidal depositions at (**a**) 6·10^16^, (**d**) 6·10^15^, and (**g**) 1.2·10^15^ NPs·cm^−3^ concentrations, and corresponding size histograms of the ZnO NWs (**b,c,e,f,h,i**). All scale bars are 4 µm.

Figure 3a shows the ZnO NWs grown using an aqueous colloidal solution of Au NPs at 6·10^12^ cm^−3^ concentration—see sample 4 in Table 2. Figure 3b–d shows the ZnO NWs obtained as a function of the Au concentration in toluene corresponding to samples 15 to 17 in Table 2. Figure 4a,d,g show the results obtained as a function of the concentration of Au NPs in hexane corresponding to samples 18 to 20 in Table 2. For all samples, the ZnO NWs density decreased with the concentration of suspended Au NPs.

The dimensions of the ZnO NWs’ diameter and length did not change with the concentration of colloidal Au NPs in water over the SiO_2_/Si substrates; see samples 4, 13, and 14 in Table 2. This involves little dependence on the concentration of the Au clusters formed from aqueous Au colloids. On the other hand, using toluene- and hexane-based Au colloids, the diameters of the ZnO NWs showed a significant variation, decreasing as the Au NP concentration decreased. As a consequence, the lengths of the ZnO NWs increased as the concentration of Au NPs decreased, as thinner NW diameters allow growing longer NW lengths in the VLS grow kinetics [28,29] see samples 15 to 20 in Table 2. The size histograms in Figure 4 show an asymmetric distribution for both diameters and lengths. This could be a direct consequence of the diffusion and coalescence of Au NPs treated at temperatures up to 900 °C, which results in an asymmetric distribution of the size of the metal nanoclusters obtained [30]. ZnO NW diameters are directly correlated with the Au nanocluster diameters; thus, ZnO NW lengths are also indirectly determined by this parameter [28,29].

Figure 3e shows a magnification image of a ZnO NW. Besides using toluene- and hexane-based colloids, in addition to the ZnO NWs, other ZnO nanostructures were obtained, such as ZnO NSs and branched nanowires (b-NWs), e.g., samples 15 to 20 in Table 2 (see Figure 3f–h). Overall, the dimensions and morphology of the ZnO nanostructures were clearly influenced by the concentration and type of the used Au colloid.

### 3.3. Au Annealing Effect

To understand the obtained results, the agglomeration with temperature of the Au NPs over the SiO_2_/Si substrates was analyzed by FESEM. Samples were annealed for 5 min in the CVD furnace at 900 °C in the absence of the ZnO/C powder mixture. Figure 3i shows results obtained from an Au colloid in water with a concentration of 6·10^12^ cm^−3^. Au domains with diameters between 40 to 60 nm and with a uniform distribution over the SiO_2_ surface were observed. Similar results were obtained for Au colloids in water at 6·10^11^ and 1.2·10^11^ NPs·cm^−3^ concentrations. The density of Au agglomerates on the SiO_2_ surface decreased with the concentration of the suspension, but the size of the clusters was maintained independently of the concentration. The thermal annealing induced aggregation of the colloidal Au NPs (19 nm) by particle migration mechanisms [31], which generated the bigger Au domains (40 to 60 nm) observed. Clusters of these sizes were found to be stable.

Figure 3j–l show the results for the deposited Au NPs using toluene as the solvent at 6·10^12^, 6·10^11^, and 1.2·10^11^ cm^−3^ concentrations, respectively. The same results were obtained for the samples using hexane as the solvent. The smaller size of the particles prepared in toluene or hexane-based colloids (11 nm) resulted in the formation of bigger Au domains compared to those obtained with aqueous colloids. The average diameter of the clusters obtained from toluene or hexane-based colloids were 140 nm and 110 nm, respectively. Furthermore, a high dispersion in cluster sizes was obtained over the SiO_2_ surface—see Figure 3j. Interestingly, aggregates with grape shapes were obtained at a concentration of 1.2·10^11^ cm^−3^ (see Figure 3l). We believe that the size of the initial Au NPs, the surface organics and the wetting of the substrate played an important role in the initial distribution of the Au NPs over the SiO_2_ surface before the annealing process, which determined the final shape of the Au domains after the thermal annealing. Furthermore, the particle size could play a role in the aggregation process at high temperatures, the larger particles being less mobile and thus resulting in clusters of smaller size.

Interestingly, ZnO NSs and b-NWs appear in a major number as the concentration of Au NPs in toluene or hexane solvents decreases. Higher concentrations of colloidal Au NPs allowed the formation of bigger Au nanoclusters after the thermal annealing, and by consequence larger and thicker ZnO nanostructures. The variety of ZnO nanostructures obtained with toluene and hexane colloids is an example of the complex Au aggregates formed over the SiO_2_ surface during the VLS growth process. An Ostwald ripening mechanism under a defocusing regime explains the large size dispersion of the obtained Au domains [31,32,33]. A lower concentration of NPs was observed to result in a larger size dispersion of Au domains, which increased the variety of ZnO morphologies grown.

### 3.4. Substrate Effect

**Figure 5:**
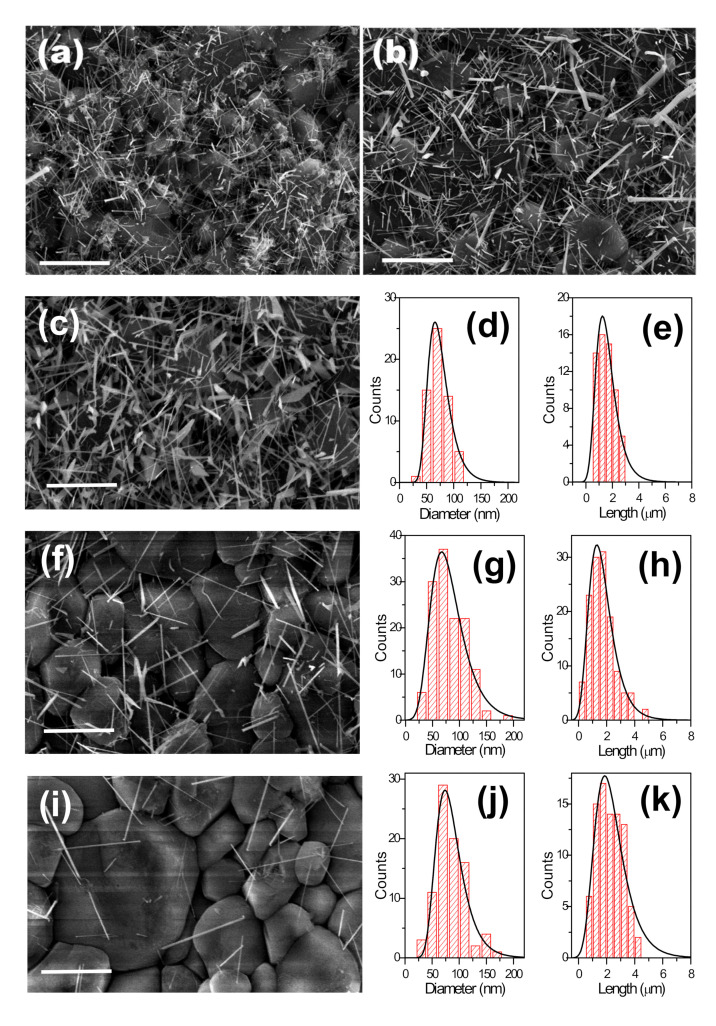
FESEM images of the ZnO NWs grown over Al_2_O_3_ substrates using (**a**) water, (**b**) toluene, and (**c**) hexane solvents for the colloidal deposition at 6·10^16^ NPs·cm^−3^ concentration; and (**f**,**i**) using hexane solvent for the colloidal depositions at 6·10^15^ and 1.2·10^15^ NPs·cm^−3^ concentrations, respectively; with corresponding size histograms of the ZnO NWs (**d,e,g,h,j,k**). All scale bars are 4 µm.

The substrate effect was analyzed with the growth of the ZnO NWs over Al_2_O_3_ substrates. Colloidal Au NPs in different solvents at 6·10^12^, 6·10^11^ and 1.2·10^11^ NPs·cm^−3^ concentrations were deposited over the Al_2_O_3_ substrates—see samples 21 to 29 in Table 2. Figure 5a–i show the results for the ZnO NWs grown using colloidal Au NPs deposited at 6·10^12^ NPs·cm^−3^ concentration in water, toluene, and hexane solvents corresponding to samples 21, 24, and 27 in Table 2, respectively.

As was also observed over the SiO_2_/Si substrates, the ZnO NWs did not grow vertically aligned but were randomly oriented due to the lack of lattice compatibility between the ZnO and the amorphous Al_2_O_3_ surface. Moreover, they were uniformly distributed over the Al_2_O_3_ substrate due to the uniform distribution of the colloidal Au NPs over the Al_2_O_3_ surface, and their density decreased with the colloidal Au NPs concentration. Figure 5c,f,i show the results obtained as a function of the concentration of Au NPs in hexane corresponding to samples 27 to 29 in Table 2.

The size histograms in Figure 5 of the ZnO NWs show an asymmetric distribution for both diameters and lengths, and the ZnO NWs density decreased with the colloidal Au NPs concentration, as was also observed over the SiO_2_/Si substrates. Surprisingly, the lengths of the ZnO NWs increased when decreasing the concentration of Au NPs while the ZnO NW diameters remained constant—see Table 2. On Al_2_O_3_ substrates, lower concentrations of colloidal Au NPs favored longer NWs due to the availability of more precursor per quantity of Au-catalyst in the VLS growth process. The ZnO NW diameters (70 nm) were larger than the initial colloidal Au NPs sizes (11 nm) because bigger Au particles were also formed at 900 °C over the Al_2_O_3_ surface. However, the diameters of the ZnO NWs did not change with colloid type and concentration over the Al_2_O_3_ substrates. This is due to the high roughness of the Al_2_O_3_ surface, which decreased the surface mobility of the deposited Au NPs in the diffusion and coalescence process [34].

### 3.5. Optical and Structural Characterization

**Figure 6:**
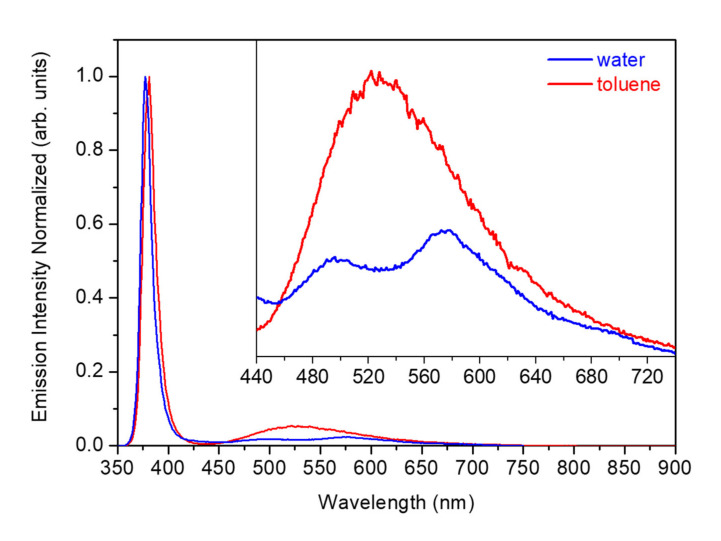
Room temperature emission spectra of the ZnO NWs grown over SiO_2_/Si substrates using water and toluene solvents for the colloidal deposition at 6·10^16^ NPs·cm^−3^ concentration.

The quantity and type of defects in the ZnO NWs can be estimated based on PL measurements [35]. Figure 6 shows the PL results of the ZnO NWs grown using the deposited Au NPs at 6·10^12^ NPs·cm^−3^ concentration in different solvents corresponding to samples 4, 15, and 18 in Table 2. The intensity of each spectrum was normalized to the intensity of the near band edge (NBE) emission for relative comparison. By pumping at 325 nm, two emission bands were observed at room temperature. A strong and narrow NBE emission in the UV at around 380 nm was associated with exciton recombination processes [36]. A broader deep level (DL) emission band in the visible range from 440 to 720 nm was related to defects [37]. The presence of a strong UV emission and a weak DL emission from the synthesized ZnO NWs indicated that the as-grown NWs had good crystal quality. The ZnO NWs obtained using a water suspension showed the UV peak at around 377 nm, and the DL broad emission band showed two peak-like features at around 500 and 580 nm. In the case of the ZnO NWs obtained using toluene or hexane solvents, the UV peak was at around 381 nm and the DL broad emission band had the maximum emission intensity at around 520 nm.

Defects responsible for the peak at around 500 nm could be attributed to zinc vacancies (V_Zn_) [38], whereas those for the peak at around 520 nm could be attributed to oxygen vacancies (V_O_) [39]. As we pointed out in a previous work [40], the peak at around 580 nm might arise from donor-acceptor-pair (DAP) recombination processes associated with Au impurities introduced during the VLS growth process [41,42]. This peak at around 580 nm has also been observed in ZnO samples intentionally doped with Au impurities [43], and in Au NPs embedded in Au:ZnO composite films [44]. The fact that the emission intensity in the visible range increased for the ZnO NWs grown using the toluene or hexane suspensions indicated that the proportion of intrinsic defects is lower for the ZnO NWs grown using the water suspension.

**Figure 7:**
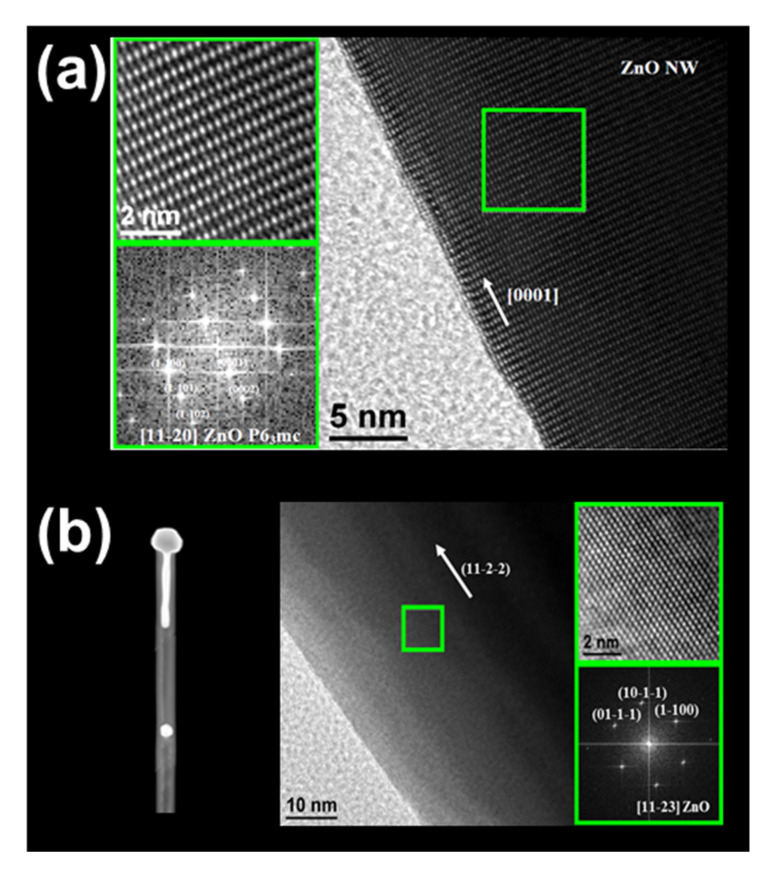
HRTEM images of a ZnO (**a**) NW and (**b**) NT in which upper panels show a high-resolution image of the area marked by the green square on the main figures, and the lower panels display the corresponding diffraction patterns.

HRTEM characterization was performed to analyze the crystal quality of the ZnO nanostructures obtained. In addition to the ZnO NWs, NSs and b-NWs observed by the FESEM characterization, HRTEM characterization demonstrated that ZnO nanotubes (NTs) were also produced. Figure 7 shows the HRTEM analysis of a ZnO NW (a) and a ZnO NT (b). As expected, the ZnO NWs showed the typical growth direction [0001], while ZnO NTs grew on the other growth direction [11-2-2]. Interestingly, the STEM image in the left part of Figure 7b shows a general view of the ZnO NT in which the Au catalyst observed at the tip diffuses inside the NT, and a drop of Au was located inside the NT.

**Figure 8:**
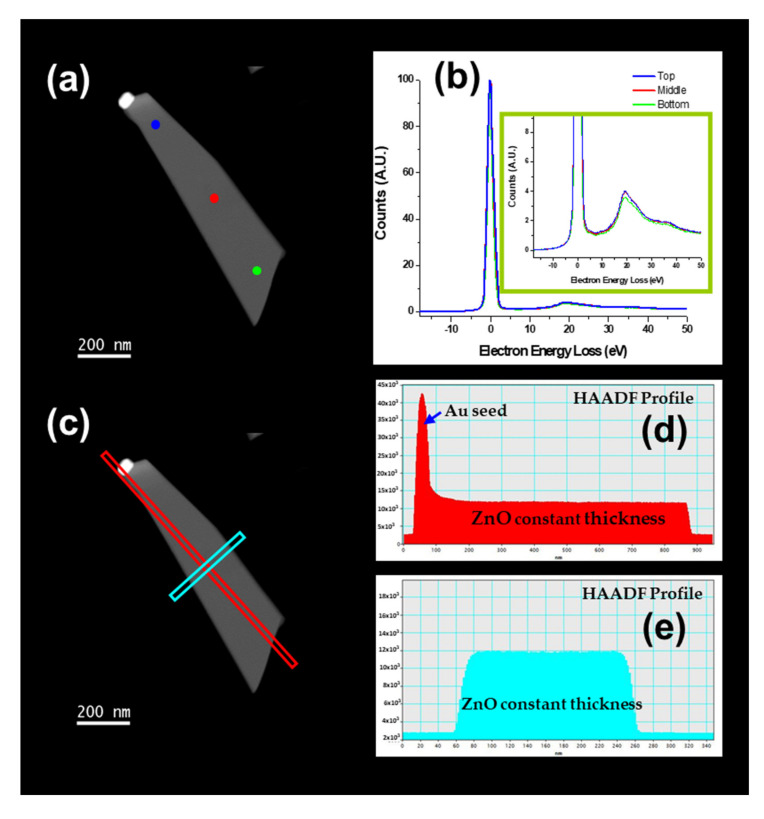
EELS spectra (**b**) and HAADF profiles (**d,e**) of a ZnO NS (**a,c**).

**Figure 9:**
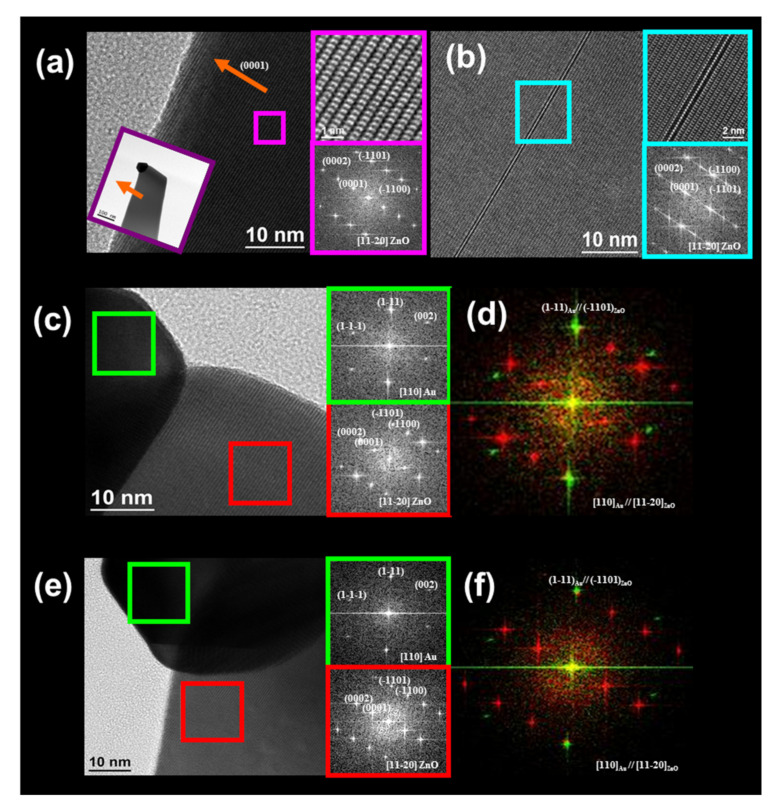
HRTEM images of a ZnO NS visualized in the zone axis [11-20]. The upper-right panels in (**a**,**b**) show a high-resolution image of the area marked by the square, and the lower-right panels display the corresponding diffraction patterns. (**c**,**e**) Show the Au/ZnO epitaxial interface. The upper-right panels show the corresponding diffraction pattern of the area marked by the green square on the Au, and the lower-right panels display the corresponding diffraction pattern of the area marked by the red square on the ZnO. (**d**,**f**) Show the corresponding diffraction patterns of the Au/ZnO epitaxial interface.

Figure 8 and Figure 9 show the HRTEM analysis of a ZnO NS. Electron energy loss spectroscopy (EELS) spectra showed that the thickness of the sheet was almost constant in all the nanostructures with a value of 47 nm, and a width of 100 to 230 nm from the top to the bottom (see Figure 8a,b). To confirm the thickness, high-angle annular dark-field (HAADF) scanning TEM measurements were performed—see Figure 8c–e. As Figure 9a shows, the most significant difference between NSs and NWs was that the growth plane of the NWs is the [0001] (see Figure 7a), while for the NSs, the [0001] growth planes are oriented laterally, in which the extension of a zone on the growth planes [0001] are transversally located. Figure 9b shows a common defect in the wurtzite hexagonal structures. Figure 9c,e show the Au/ZnO epitaxial interface. The characteristic diffraction patterns presented in Figure 9d,f show the epitaxial relation (1-11)[110]Au//(-1101)[11-20]_ZnO_ in the Au and ZnO areas of the NS.

**Figure 10:**
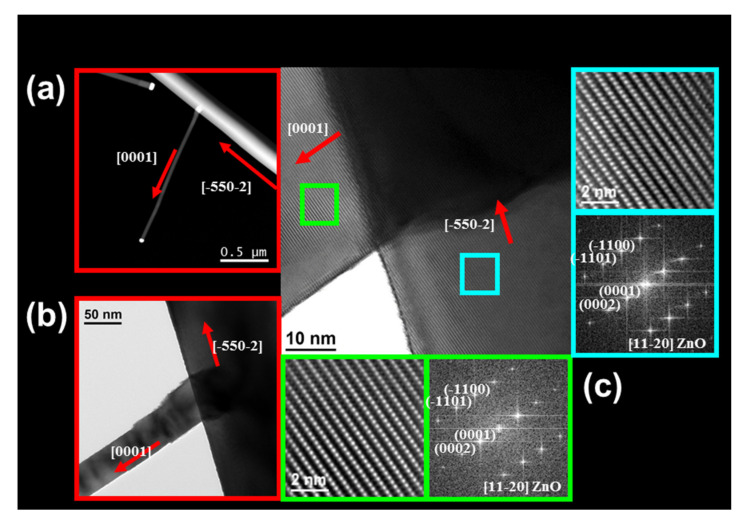
HRTEM images of a ZnO b-NW visualized in the zone axis [11-20]. (**a**,**b**) Show the growth directions marked with red arrows. (**c**) Shows the ZnO NW/b-NW epitaxial interface. The upper-right panel shows the high-resolution image of the area marked by the blue square on the NW and the lower-right panel displays the corresponding diffraction pattern. The bottom-left panel shows the high-resolution image of the area marked by the green square on the b-NW and the bottom-right panel displays the corresponding diffraction pattern.

Figure 10 shows the HRTEM analysis of a ZnO b-NW. The branches grow along the most favorable direction [0001] from a ZnO NW that grows along the unusual growth direction [-550-2], with a perfect epitaxial relation between the NW and the branches—see the red arrows in Figure 10. The branch length depends on the position of the branch along the ZnO NW; the branches nearer to the base are longer and the length decreases linearly as they approach the ZnO NW tip where the Au particle is located and the ZnO growth takes place (see Figure 3g,h). In addition, the distribution of the branches is not random but forms a straight line along the main ZnO NW, appearing to have between four and seven branches in a distance from 1.5 to 5 µm.

### 3.6. Nanostructures Growth Mechanisms

**Figure 11:**
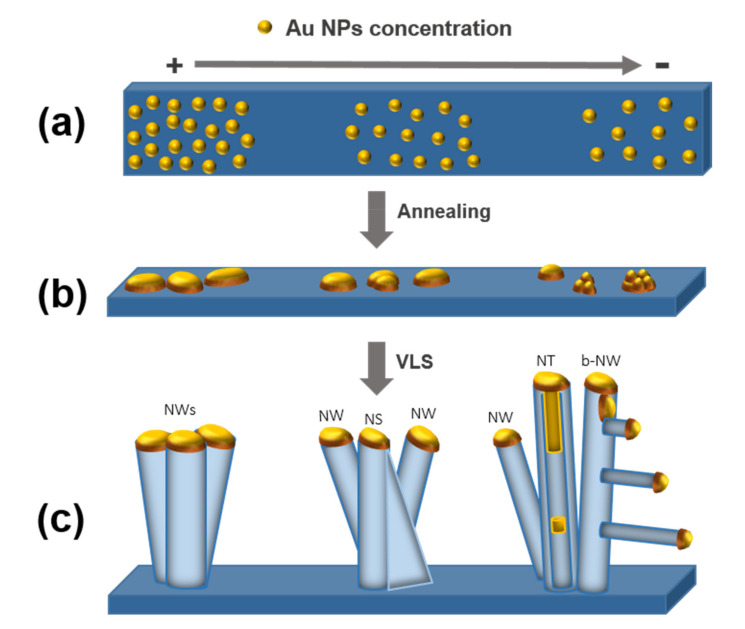
Growth mechanisms proposed for the obtained ZnO nanostructures over the SiO_2_/Si substrates. (**a**) Au NPs deposition, (**b**) thermal annealing, and (**c**) VLS growth of ZnO nanostructures.

Taking into account the results presented, Figure 11 proposes the growth mechanisms for the ZnO nanostructures obtained over the SiO_2_/Si substrates. There are three key steps: (a) Au NPs deposition, (b) thermal annealing, and (c) VLS growth of ZnO.

(a) *Au NPs deposition*. Figure 11a represents the Au NPs deposited over the SiO_2_ surface. In previous work, we reported that the solvent used in the suspensions influences the NPs’ distribution over the surface [35]. The chemical interactions of the solvent with the colloidal Au NPs and the surface determine their mobility and coalescence during the deposition step. Additionally, it was demonstrated that using an organic solvent promotes a spontaneous self-assembly of colloidal Au NPs covered with weak aliphatic ligands (i.e., decanoic acid) [45]. In the case of a polar solvent such as water, the repulsive interactions of the water solvent with the aliphatic surface and the slow solvent evaporation over the wettable SiO_2_ surface enhance the proximity of the Au NPs deposited in small aggregates over the surface. On the contrary, non-polar organic solvents such as toluene or hexane were characterized by a faster evaporation and lower wettability on the SiO2 surface, thus generating Au NP aggregates with a large size distribution.

(b) *Thermal annealing*. In this step, the diffusion and coalescence of the deposited Au NPs takes place (see Figure 11b). Theoretical studies show that the coalescence depends on the temperature and proximity of the colloidal Au NPs distributed over the surface. Higher temperatures than 700 °C force the particle migration. When the distance between the colloidal Au NPs is close, the van der Waals forces are higher than Pauli repulsive forces, promoting the diffusion and coalescence of the Au NPs [19]. Homogenous NP distributions result in clusters with homogeneous distribution and diameters, as observed with aqueous colloids (see Figure 3i). On the other hand, the inhomogeneous distribution of NPs obtained from toluene or hexane colloids and a defocusing Ostwald ripening resulted in Au agglomerates with a large size and shape dispersion (see Figure 3j–l). Carbon produced by the calcination of the organic ligands covered the surface of the Au NPs during the calcination. This carbon could be responsible for modifying the coalescence process, preventing a complete coalescence of the Au in spherical Au particles, but forming aggregates with a variety of shapes. This effect is more notorious for toluene- or hexane-based colloids, having a larger amount of organics on their surface. For all these reasons, the Au colloid used to distribute Au over the SiO_2_ surface during the deposition process plays an important role in how the final shape of the obtained Au clusters at different concentrations will be (i.e., spherical, elliptical, or agglomerated as irregular shapes) (see Figure 3j–l).

(c) *VLS growth of ZnO*. The last step consists of the growth of the ZnO nanostructures—see Figure 11c. At this point, the Au-catalyst is enriched with Zn atoms from the carbothermal reduction. At high concentrations of colloidal Au NPs, the ZnO NWs grow following the VLS growth mechanism. ZnO NSs also grow following the VLS growth mechanism, while the vapor-solid (VS) process is responsible for the horizontal growth direction of the sheet. Then, over the surface of the sheet, the resulting ZnO molecules will attach to an energetically favorable site, forming the ZnO NS. At low concentrations of colloidal Au NPs, the incomplete coalescence of Au NPs resulting in grape shapes leads to the defragmentation of the Au catalyst inside the wire generating a ZnO NT (see Figure 7b), or outside in the case of the ZnO b-NW (see Figure 10a). The length of the ZnO b-NWs depends on the position of the branch along the main ZnO NW; the branch length decreases as it gets closer to the tip because the growth time decreases. ZnO b-NWs grew following the VLS growth mechanism, in which the Au-catalyst of the main ZnO NW is defragmented and decanted over the walls favoring the growth of the branches—see Figure 3g,h.

## 4. Conclusions

The use of colloidal Au NPs as catalysts for the VLS growth of ZnO NWs is not only a low-cost alternative to the conventional processing methods, but it also allows a tuning of the nanostructure characteristics. The solvent and the concentration of the colloidal Au NP solutions not only determine their density but also have a strong influence on their dimensions and morphology. Furthermore, the substrate plays a significant role in determining the diameter of the ZnO NWs. Such a role is associated with the substrate influence on the initial distribution of NPs and on the surface mobility of the deposited colloidal Au NPs at high temperatures. ZnO NWs were the only product of the process when using an aqueous suspension of Au NPs, while using toluene or hexane solvents ZnO NWs, NSs, NTs, and b-NWs were produced. The ZnO nanostructure variety increased when decreasing the colloidal Au NPs concentration.

## Figures and Tables

**Table 1:** Experimental details of the VLS assisted growth parameters and of the dimensions of the ZnO NWs using water solvent grown over SiO_2_/Si substrates.

Sample Number	Growth Parameter and Dimensions of the ZnO NWs				
	Temperature (°C)	Pressure (Torr)	Ar Gas Flow (sccm)	Diameter (nm)	Length (µm)
1	700	760	400	-	-
2	800	760	400	-	-
3	850	760	400	70 ± 10	0.7 ± 0.1
4	900	760	400	100 ± 10	2.8 ± 0.2
5	900	76	400	-	-
6	900	200	400	-	-
7	900	380	400	100 ± 10	1.3 ± 0.1
8	900	600	400	120 ± 10	2.3 ± 0.2
9	900	760	50	-	-
10	900	760	100	120 ± 10	3.7 ± 0.2
11	900	760	200	140 ± 10	3.4 ± 0.2
12	900	760	600	100 ± 10	2.7 ± 0.2

**Table 2:** Experimental details of the colloidal Au NPs and dimensions of the ZnO NWs.

Sample Number	Colloidal Au NPs		ZnO NWs		
	Solvent	Concentration (NPs·cm^−3^)	Substrate	Diameter (nm)	Length (µm)
4	water	6.1·10^16^	SiO_2_/Si	100 ± 10	2.8 ± 0.2
13	water	6.1·10^15^	SiO_2_/Si	100 ± 10	2.8 ± 0.2
14	water	1.2·10^15^	SiO_2_/Si	100 ± 10	2.8 ± 0.2
15	toluene	6.1·10^16^	SiO_2_/Si	150 ± 10	2.0 ± 0.2
16	toluene	6.1·10^15^	SiO_2_/Si	120 ± 10	2.2 ± 0.2
17	toluene	1.2·10^15^	SiO_2_/Si	100 ± 10	2.5 ± 0.2
18	hexane	6.1·10^16^	SiO_2_/Si	160 ± 10	1.9 ± 0.2
19	hexane	6.1·10^15^	SiO_2_/Si	140 ± 10	1.8 ± 0.2
20	hexane	1.2·10^15^	SiO_2_/Si	100 ± 10	2.2 ± 0.2
21	water	6.1·10^16^	Al_2_O_3_	70 ± 10	0.8 ± 0.2
22	water	6.1·10^15^	Al_2_O_3_	70 ± 10	1.0 ± 0.2
23	water	1.2·10^15^	Al_2_O_3_	70 ± 10	1.4 ± 0.2
24	toluene	6.1·10^16^	Al_2_O_3_	70 ± 10	1.4 ± 0.2
25	toluene	6.1·10^15^	Al_2_O_3_	70 ± 10	1.6 ± 0.2
26	toluene	1.2·10^15^	Al_2_O_3_	70 ± 10	2.2 ± 0.2
27	hexane	6.1·10^16^	Al_2_O_3_	70 ± 10	1.3 ± 0.2
28	hexane	6.1·10^15^	Al_2_O_3_	70 ± 10	1.5 ± 0.2
29	hexane	1.2·10^15^	Al_2_O_3_	70 ± 10	2.0 ± 0.2
